# Sepsis-Induced Acute Lung Injury (ALI) Is Milder in Diabetic Rats and Correlates with Impaired NFkB Activation

**DOI:** 10.1371/journal.pone.0044987

**Published:** 2012-09-14

**Authors:** Luciano R. Filgueiras, Jr., Joilson O. Martins, Carlos H. Serezani, Vera L. Capelozzi, Marlise B. A. Montes, Sonia Jancar

**Affiliations:** 1 Department of Immunology, Institute of Biomedical Sciences, University of São Paulo, São Paulo, Brazil; 2 Department of Clinical and Toxicological Analyses, Faculty of Pharmaceutical Sciences, University of São Paulo, São Paulo, Brazil; 3 Department of Microbiology and Immunology, Indiana University School of Medicine, Indianapolis, Indiana, United States of America; 4 Department of Pathology, Faculty of Medicine, University of São Paulo, São Paulo, Brazil; Fundação Oswaldo Cruz, Brazil

## Abstract

Acute lung injury (ALI) develops in response to a direct insult to the lung or secondarily to a systemic inflammatory response, such as sepsis. There is clinical evidence that the incidence and severity of ALI induced by direct insult are lower in diabetics. In the present study we investigated whether the same occurs in ALI secondarily to sepsis and the molecular mechanisms involved. Diabetes was induced in male Wistar rats by alloxan and sepsis by caecal ligation and puncture surgery (CLP). Six hours later, the lungs were examined for oedema and cell infiltration in bronchoalveolar lavage. Alveolar macrophages (AMs) were cultured in vitro for analysis of IκB and p65 subunit of NFκB phosphorylation and MyD88 and SOCS-1 mRNA. Diabetic rats were more susceptible to sepsis than non-diabetics. In non-diabetic rats, the lung presented oedema, leukocyte infiltration and increased COX2 expression. In diabetic rats these inflammatory events were significantly less intense. To understand why diabetic rats despite being more susceptible to sepsis develop milder ALI, we examined the NFκB activation in AMs of animals with sepsis. Whereas in non-diabetic rats the phosphorylation of IκB and p65 subunit occurred after 6 h of sepsis induction, this did not occur in diabetics. Moreover, in AMs from diabetic rats the expression of MyD88 mRNA was lower and that of SOCS-1 mRNA was increased compared with AMs from non-diabetic rats. These results show that ALI secondary to sepsis is milder in diabetic rats and this correlates with impaired activation of NFκB, increased SOCS-1 and decreased MyD88 mRNA.

## Introduction

Diabetes is a syndrome characterized by chronic hyperglycaemia with disturbances in protein, lipid and carbohydrate metabolism owing to a deficiency in insulin production, action or both. In type 1 diabetes (T1D) the patients exhibit defective insulin production [1].

In experimental models of T1D, many aspects of the inflammatory response are reduced such as leukocytes' adhesion to endothelium and migration into the inflammatory site, mast cell degranulation, and production of prostaglandin (PG) E_2_ [2]. Moreover, the phagocytes from diabetic rats have reduced capacity to ingest fungi [2] and IgG opsonized targets [3] and bacterial clearance is reduced in the peritoneal cavity of mice submitted to colon ligation and puncture (CLP) [4]. These alterations in inflammation and innate immunity contribute to the increased susceptibility to infection of diabetics.

In clinical studies, it was reported that the incidence of sepsis is increased in diabetic patients [5], [6]. Sepsis develops when the initial host response to an infection is amplified and becomes damaging to the host [7]. Some structural components of bacteria (pathogen-associated molecular patterns - PAMPs), are recognized by pattern recognition receptors (PRRs) expressed in phagocytes and other cell types [8] and are responsible for the initiation of the septic process. Upon infection with gram-negative bacteria, lipopolysaccharide (LPS) has a central role in disease development. The receptor complex formed by toll-like receptor (TLR) 4 and CD14 constitutes the LPS receptor in the host cells [8], and the signalling programme is initiated by two major distinct pathways: the myeloid differentiation factor 88 (MyD88) and the TIR-domain-containing adapter-inducing IFN-β (TRIF) pathway. Both pathways result in activation of NFκB and transcription of several pro-inflammatory genes [8].

This amplified response, also called cytokine storm, results in a systemic inflammation that affects several organs. The lung is particularly affected and acute lung injury (ALI) secondary to sepsis is characterized by oedema, inflammatory cell infiltration and, in consequence, impaired gas exchange. In its severe form, hypoxia aggravates the patient´s condition and can lead to multi-organ failure [9]. About 40% of septic patients develop ALI [10]. In diabetics, however, the incidence of ALI is much lower [11] and respiratory failure is less frequent [12]. Thus, diabetes seems to exert a protective role in ALI although the mechanisms of this ‘protection’ are still unknown.

**Table 1:** Sequence of primer pairs used in semi-quantitative real-time PCR.

Genes	Right	Left
MyD88	5′-GATAGGCATGTCAGGGGAGA-3′	5′-GCTGACTTGGAGCCTGATTC-3′
SOCS-1	5′-GAAGGTGCGGAAGTGAGTGT-3′	5′-TGGTAGCACGTAACCAGGTG-3′
GAPDH	5′-GCCAGCCTCGTCTCATAGACA-3′	5′-TGGTAACCAGGCGTCCGATA-3′

In the present study we compared sepsis-induced ALI in diabetic and non-diabetic rats and investigated the molecular mechanisms that regulate the development of ALI. To this purpose we used the alloxan-induced diabetes that is extensively used as a model for T1D and the CLP model of sepsis which resembles the bacterial dissemination seen in human infectious sepsis [13].

**Table 2:** Blood glucose levels (mg/dL) in diabetic* and non-diabetic rats and effect of sepsis induced by CLP**.

	Non-diabetic		Diabetic	
	0 h	6 h	0 h	6 h
SHAM	96,6±6,6	105,2±5,3	534,2±27,8	468,6±35,3
CLP	92,4±3,1	91,2±3,5	517,0±36,8	438,7±19,9

* Alloxan (42 mg/Kg) was given i.v. and glucose levels were determinate 10 days later.

** CLP – Colon Ligation and Puncture (20 G needle –12 punctures).

## Materials and Methods

### Animals

Specific pathogen-free male Wistar rats weighing 200±20 g at the beginning of the experiments were used. The animals were maintained at 23°C under a 12-h light-dark cycle and were allowed access to food and water ad libitum. This study was carried out in strict accordance with the principles and guidelines adopted by the Brazilian College of Animal Experimentation and approved by the Ethical Committee for Animal Research of the Biomedical Sciences Institute, University of São Paulo (Permit Number: 139-65-02). All surgery was performed under ketamine anaesthesia, and all efforts were made to minimize suffering.

**Figure 1:**
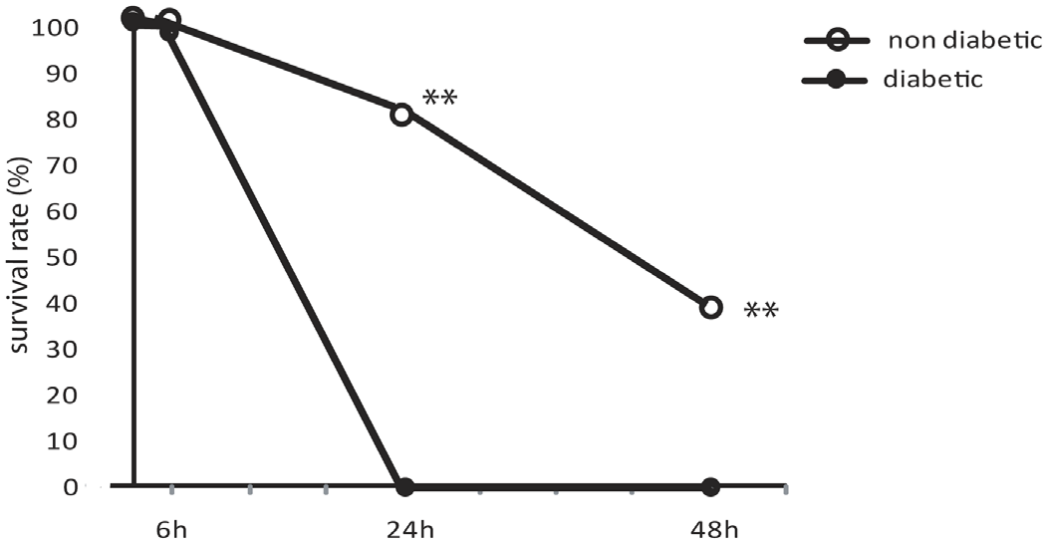
Survival rate. Percentage of diabetic and non-diabetic rats submitted to CLP-induced sepsis that died in a given time. Diabetes was induced by i.v. injection of alloxan (42 mg/kg/iv) in Wistar rats and 10 days later CLP was performed (12 punctures with a 20 G needle). n = 5/group, repeated three times with identical results. Data are presented as mean ± SEM. **P<0.01 diabetic vs. non-diabetic.

### Alloxan-induced Diabetes(T1D)

T1D was induced by alloxan injection (42 mg/kg, i.v.), as previously described by Martins et al. [9]. After 10 days, the glycaemia of the injected animals was measured with Accu-Chek Advantage II (Roche Diagnostica, Sao Paulo, SP, Brazil). Only animals presenting glycaemia >200 mg/dL were considered as diabetic for the purposes of this research. The control group (non-diabetic animals) was injected with the same volume of the vehicle (NaCl, 0.9%).

**Figure 2:**
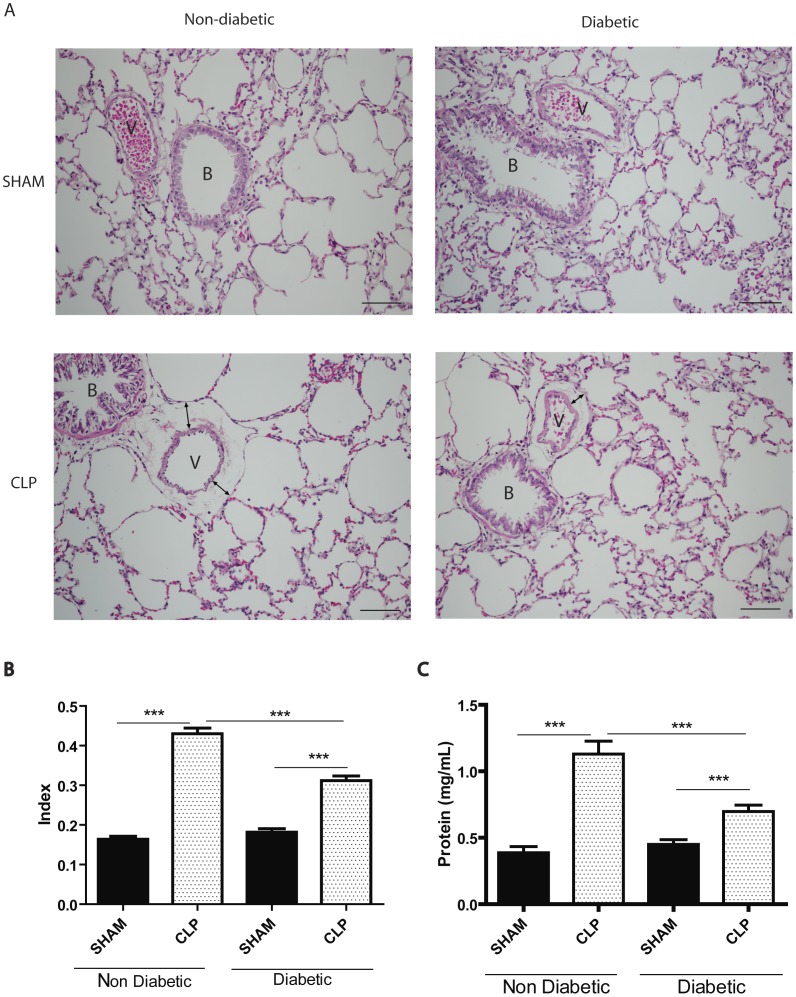
Lung oedema at 6 h after CLP. Diabetic and non-diabetic rats were submitted to CLP and after 6 h the lungs were removed and processed. (**A**) Photomicrographs of peribronchovascular axis in lung stained with haematoxylin-eosin; ‘**B**’ stands for bronchiole and ‘**V**’ for venule. Note the presence of oedema around the venule (leakage area marked in black bars). Photographs were taken at an original magnification of 200x. (**B**) Quantification of perivascular oedema by light microscopy with an integrating eyepiece with a coherent system consisting of a grid with 100 points and 50 lines (known length).The number of points falling on areas of perivascular oedema and the number of intercepts between the lines of the integrating eyepiece and the basal membrane of the vessels were counted. The oedema index was calculated as follows: number of points^1/2^/number of intercepts. Ten random non-coincident microscopic fields containing a bronchus and a venule were evaluated for each group, n = 5 per group. (**C**) Evaluation of lung oedema by total protein content in the BAL after 6 hours of CLP or sham-operated, n = 5/group and scale bar  = 50 µm. Data are presented as mean ± SEM. ***P<0.001.

### Sepsis-induced ALI

The animals were anaesthetized by an intraperitoneal injection (150 mg/kg) of ketamine hydrochloride (Ketamin-S(+); Cristalia, São Paulo, Brazil), a midline laparotomy was performed, the caecum was exposed, ligated and punctured 12 times with a 20-gauge needle. The caecum was replaced in the abdomen, and the incision was closed. Another group of rats was subjected to midline laparotomy and manipulation of the caecum without ligation and puncture (sham operation). After the surgery, the animals were returned to their cages and were allowed access to food and wate*r* ad libitum. Six hours after CLP, the animals were anaesthetized, as described previously, and exsanguinated from the abdominal aorta. For bronchoalveolar lavage (BAL), 10 mL of phosphate-buffered saline (PBS) was instilled intra-tracheally, and the recovered sample was centrifuged (500Xg for 15 min). Protein concentration was determined in the BAL supernatant, as a measure of oedema, by means of a commercially available kit (BCA™ Protein Assay Kit, Pierce Biotechnology Inc., Rockford, IL, USA). The pellet was re-suspended in PBS, and total cell counts were performed under light microscopy. Differential cell counts were carried out on haematoxylin-eosin stained preparations under oil immersion microscopy. In another set of experiments, the lungs were removed 6 h after CLP, rinsed and the lobulated side immediately immersed in 10% buffered formaline for histopathologic and morphometric analysis, and the other side was stored in liquid nitrogen until processed for Western blot analysis. For survival rate determination, five animals were used in each group and this was repeated three times.

**Figure 3:**
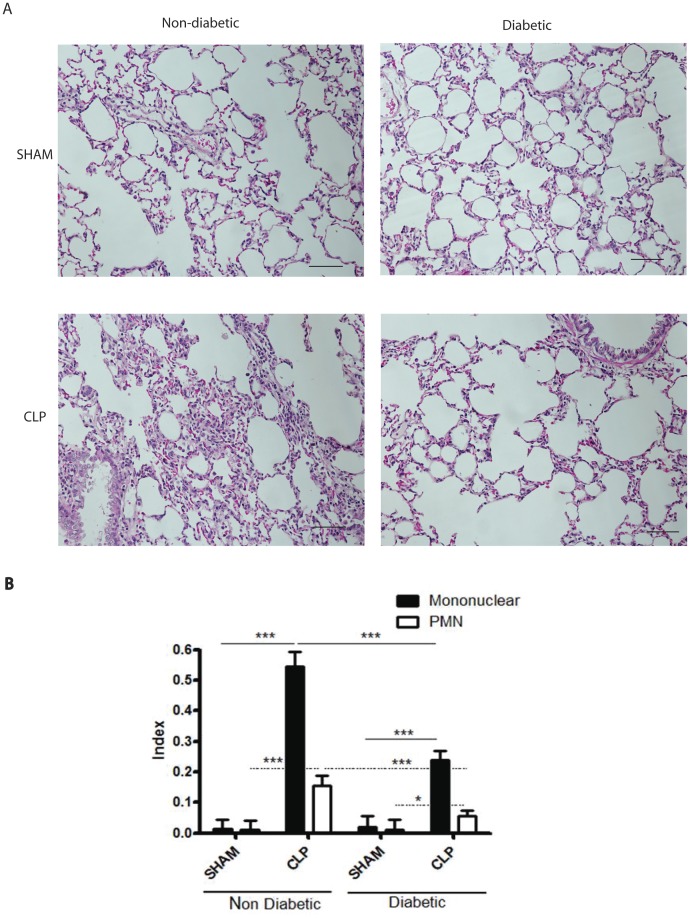
Inflammatory cell infiltration in lung at 6h after CLP. Diabetic and non-diabetic rats were submitted to CLP and after 6h the lungs were removed and processed. (**A**) Photomicrographs of lung parenchyma stained with haematoxylin-eosin. (**B**) Mononuclear and polymorphonuclear cell index was determined in the parenchyma. The cell index quantification was performed with an integrating eyepiece with a coherent system consisting of a grid with 100 points and 50 lines (known length); cells were evaluated at x1,000 magnification. Points falling on mononuclear or PMN cells were counted and divided by the total number of points falling on tissue areas in each microscopic field. Ten random non-coincident microscopic fields were evaluated for each group, n = 5/group and scale bar  = 50 µm. Data are presented as mean ± SEM. * P<0.05; ***P<0.001.

### Immunoblotting

Lungs were homogenized in PBS containing 1% of protease inhibitor cocktail according to the manufacturer’s instructions (Sigma Chemical Co, St. Luis, MO, USA.). Samples containing 20 µg protein were separated by sodium dodecyl sulfatepolyacrylamide gel electrophoresis (10%) and transferred to nitrocellulose membrane (Invitrogen, Carlsbad, CA, USA ). The membranes were incubated in TSB-T (150 mM NaCl, 20 mM Tris, 1% Tween 20, pH 7.4) containing 5% non-fat dried milk for 60 min. After that, the blots were washed with TSB-T and probed with antibodies (1∶500 diluted; Cayman Chemical, Ann Arbor, MI, USA) against cyclooxygenase (COX)- 2; (1∶500 diluted; Abcam) and against phospho-IkB-α for 120 min at room temperature. The membranes were washed with TBS-T and incubated with peroxidase-conjugated monoclonal anti-rabbit immunoglobulin G (1∶2000) for 60 min at room temperature. The immuno-complexed peroxidase-labelled antibodies were visualized by an enhanced chemiluminescence (ECL) kit following the manufacturer’s instructions (Amersham, Piscataway, NJ, USA) and exposed to photographic films. Finally, blots were stripped and reprobed for β-actin. The band densities were determined by densitometric analysis by means of the AlphaEaseFCi program (Alpha Innotech, San Leandro, CA, USA ). Density values of bands were normalized to the total β-actin present in each lane and expressed as percentage of control.

**Figure 4:**
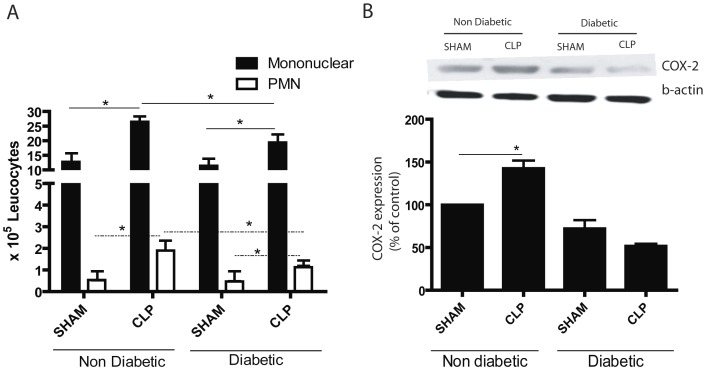
Inflammatory cell infiltration in BAL fluid and lung COX2 expression at 6h after CLP. Diabetic and non-diabetic rats were submitted to CLP or SHAM (false surgery) and after 6h bronchoalveolar lavage (BALF) was performed. (**A**) mononuclear and PMN cell were counted in haematoxilin-eosin stained cytospin preparations of BALF cells after total cell count was performed under light microscopy. (**B**) Expression of COX2 protein in lung homogenates six hours after CLP analysed by immunoblotting using antibodies to COX-2 and quantified by densitometric analysis of the immunoblot bands. Density values of bands were normalized to the total β-actin present in each lane and expressed as a percentage of control. n = 5/group, data are presented as mean ± SEM; *, P<0.05.

### Isolation of Alveolar Macrophage

Rat AMs were obtained by lung lavage and allowed to adhere in culture plates for 1 h (37°C, 5% CO2); this was followed by two washes with warm RPMI, resulting in more than 99% adherent cells identified as AMs, as described previously [14](n = 5 per group).

**Figure 5:**
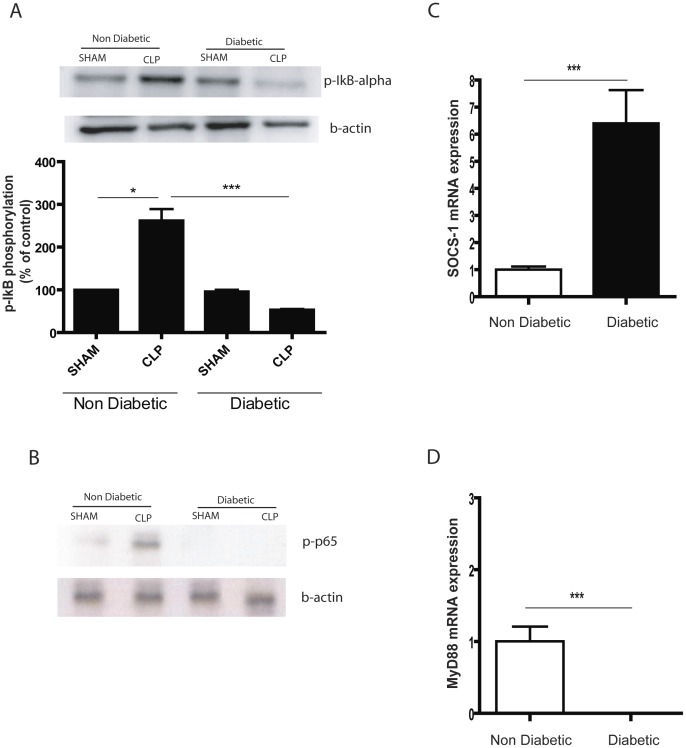
NFkB activation in alveolar macrophages 6h after CLP. Alveolar macrophages **(**AM) were obtained by lung lavage six hours after CLP and allowed to adhere in culture plates for 1 h. Total mRNA or total protein was extracted from AMs. (**A**) 20 µg of total protein analysed by immunoblotting using antibodies to phosphorylated – IκBα and β-actin.The bands were quantified by densitometric analysis. Density values of bands were normalized to the total β-actin present in each lane and expressed as percentage of control. (**B**) 50 µg of total protein analysed by immunoblotting using antibodies to phosphorylated – p65 and β-actin. (**C**) cDNA was synthesized from total mRNA extracted and the expression of SOCS-1. (**D**) MyD88 mRNA was analysed by RT-PCR. mRNA expression levels were calculated by the comparative Ct method and normalized to GAPDH levels with non-diabetic CLP given an arbitrary value of one. n = 5/group, data were presented as mean ± SEM.* P<0.05; ***, P<0.001.

### Real-time PCR

Total RNA was extracted with TRIzol Reagent (Invitrogen, USA) and the concentration of RNA was determined by spectrophotometer readings at absorbance 260 nm. cDNA were synthesized by RevertAid First strand (Fermentas life sciences, Ontario, USA). Real-time PCR was performed with semi-quantitative SYBR Green assay (Applied Biosystem, USA) using specific primers for MyD88 and SOCS-1 (Table 1).

The amount of the target gene was normalized first to the endogenous reference (GAPDH) and then relative to a calibrator (sample with the lowest expression - control animal); data were analysed by the comparative C(T) method [15]. Hence, steady-state mRNA levels were expressed as an n-fold difference relative to the calibrator. Analyses were performed with the MxPro- Mx3005P v3.00.

### Histology

Lungs were dehydrated in 70% ethanol, processed using standard procedures and embedded in paraffin. Sections of 5 µm were cut, mounted on slides, and stained with haematoxylin and eosin. The histopathology analysis was performed with a conventional light microscope (Olympus BX51, Olympus Latin America, São Paulo, Brazil) and images were captured with a Nikon DXM1200C digital camera.

### Lung Morphometric Analysis for Cell Infiltration and Oedema

Lung morphometric analysis was performed with an integrating eyepiece with a coherent system consisting of a grid with 100 points and 50 lines (known length) coupled to a conventional light microscope (Olympus BX51, Olympus Latin America, São Paulo, Brazil). Polymorphonuclear and mononuclear cells were evaluated at x1,000 magnification, and 10 random, non-coincident microscopic fields were evaluated for each group, n = 5 per group. Points falling on polymorphonuclear and mononuclear cells were identified by conventional morphology, counted and divided by the total number of points falling on the tissue area in each microscopic field as described in [16]. To quantify interstitial oedema, 10 random non-coincident microscopic fields containing a bronchus and a venule were evaluated for each group, n = 5 per group. The number of points falling on areas of perivascular oedema and the number of intercepts between the lines of the integrating eyepiece and the basal membrane of the vessels were counted. The interstitial perivascular oedema index was calculated as follows: number of points^1/2^/number of intercepts [17].

### Statistical analysis

Data were presented as mean ± standard error of the mean (SEM) and were analysed by Student t test or ANOVA followed by the Tukey-Kramer multiple comparisons test when appropriate. P<0.05 was considered significant. Survival rates were analysed with the log-rank test.

## Results

### Diabetic Rats are More Susceptible to Sepsis

Diabetes was induced by alloxan injection (42 mg/kg, i.v.) and 10 days later the blood glucose was measured, being under 100 mg/dL in non-diabetic rats and over 300 mg/dL in diabetic rats.

Sepsis was induced by 12 caecal punctures (CLP) and did not affect the blood glucose levels (Table 2). CLP was followed by a significant reduction in blood leukocytes in both diabetic and non-diabetic groups (from 13.9±1.0 to 6.2±0.8 in diabetics and from 13.4±1.0 to 7.2±1.2×10^3^ in non-diabetics) whereas the sham surgery did not affect the number of blood leukocytes in diabetic and non-diabetic rats (from 12.9±1.5 to 13.1±1.4 in diabetics and 13.2±1.1 to 12.9±1.1×10^3^ in non-diabetics).

After 24 h of sepsis induction all diabetic animals were dead whereas 80% of the non-diabetic rats were still alive (Figure 1). In the present study we evaluated the lung inflammation after 6 h of sepsis induction since this was the last point at which 100% of the animals were still alive.

### ALI Secondary to Sepsis is Milder in Diabetic Rats

Sepsis induces acute lung inflammation that, depending on severity, can impair gas exchange leading to hypoxia and multi-organ failure. Since the diabetic rats were more susceptible to sepsis, we sought to investigate whether this greater susceptibility leads to more severe ALI.

Figure 2A shows the lungs of CLP and sham-operated non-diabetic and diabetic rats, stained by haematoxylin and eosin. The illustration shows a bronchiole with a small blood vessel. After 6 h of CLP induction, oedema can be seen around the blood vessel in both groups. At higher magnification, the lungs from diabetic rats showed intra-alveolar oedema and the alveolar septa were thicker (data not shown). Histomorphometric analysis confirmed the pattern observed in Figure 2A. The oedema area around 10 blood vessels from each lung (n = 5/group) was measured and the index was calculated as described in MM. Figure 2B shows that the non-diabetic group with CLP exhibits a markedly increased index, contrasting with the sham-operated group. The diabetic group with CLP also exhibited increased index when compared with the sham-operated. Comparison of the CLP groups showed that the oedema index was significantly lower in the diabetics. This was confirmed by measuring the protein content in the bronchoalveolar lavage fluid. Figure 2C shows an increase in protein concentration in the CLP group compared with the sham-operated. In diabetics with CLP the protein levels were lower, indicating that they had developed less oedema than the non-diabetic septic rats.

Figure 3A shows that 6h after induction of CLP there was a strong recruitment of leukocytes into the lung parenchyma, evaluated by morphometric analysis in histological preparations. The cell infiltrate was of mono and polimorphonuclear cells but mononuclear cells predominated. Although the pattern of cell infiltration was similar in diabetic and non-diabetic rats, in diabetic rats the number of infiltrated cells was significantly lower (Figure 3B). Figure 4A shows that following CLP there was also a marked influx of inflammatory cells into the bronchoalveolar space which was significantly lower in diabetic rats.

When expression of the inducible enzyme COX-2 (which generates prostanoids, including PGE_2_) was measured, it had also increased after CLP (Figure 4B). In diabetic rats, the level of COX-2 did not change after CLP.

### Alveolar Macrophages from Diabetic Rats with Sepsis have Impaired NFκB Activation

The observation that lung injury was milder in diabetic rats with sepsis prompted us to investigate whether this was owed to an impaired response of AMs to stimuli derived from the systemic infection. AMs were obtained by bronchoalveolar lavage performed after 6h of sepsis induction.

NFκB exists in unstimulated cells as a transcriptional dimer (p50 and p65 subunits) sequestered in the cytoplasm by the inhibitor protein IκB-α. Upon cell activation IκB-α is phosphorylated and degraded, releasing NFκB subunits which allows NFκB to translocate to the nucleus and promote transcription of target genes. We first investigated the phosphorylation of the regulator protein IκB-α and found that the phosphorylation of IκB-α occurred only in AMs from the septic non-diabetic group, indicating that in diabetic cells the NFκB activation is impaired (Figure 5A). This was confirmed when we analysed the phosphorylation of the transcriptional subunit p65. The phosphorylation of p65 increases NFκB interaction with the co-activator p300/CBP and enhances the transcriptional activity of this factor. It was found that p65 was phosphorylated in AMs from septic non-diabetic rats but not in those from diabetic rats (Figure 5B). These results suggest that AMs from diabetic rats are unable to activate NFκB during sepsis.

It is known that LPS induces NFκB activation in a MyD88-dependent manner during sepsis [18] and that the expression of this adaptor protein is negatively regulated by SOCS-1 [19] so we then investigated the MyD88 and SOCS-1 (Suppressor of Cytokine Signalling) expression in the septic animals. It was found that AMs from diabetic rats with sepsis express higher levels of SOCS1 mRNA compared with non-diabetics (Figure 5C). The inverse was found for MyD88 mRNA, which was expressed in non-diabetic CLP rats but not detected in diabetic septic rats (Figure 5D).

Thus in AMs from diabetic rats with sepsis, the enhanced expression of the molecular brake SOCS-1 decreases MyD88 expression and therefore NFκB activation does not occur. This could explain the milder sepsis-induced ALI in diabetic rats.

## Discussion

In the present study, diabetes was induced by Alloxan, a diabetogenic drug that induces the production of reactive oxygen species (ROS) that accumulate in the pancreatic islets causing an irreversible lesion of the cells responsible for insulin synthesis, the β cells [20]. The CLP model is one of the most widely used models of sepsis and septic shock and resembles human sepsis in many parameters [13]. In this model, the severity of sepsis correlates with the number of colon punctures. We tested 4, 8 and 12 punctures and since our focus was on the lung inflammation secondary to sepsis, we chose to use 12 punctures to ensure measurable alterations in the lung. By choosing this protocol, however, we were limited by time, as 6 h after CLP was the maximum time point when all animals subjected to 12 punctures were still alive. After 24 h, all the diabetic rats were dead compared with 20% of the non-diabetics.

ALI can be divided into two forms depending on the origin of the insult. The extra-pulmonary form occurs secondarily to a systemic process and the pulmonary form occurs when the injury is primarily to lung parenchyma [10]. In our model of extra-pulmonary ALI, after 6 h of CLP induction we observed the formation of peribronchovascular oedema, intra-alveolar oedema, septa lungs thickened by capillary congestion and cell infiltration in the lung. Although polymorphonuclear cells were present, the mononuclear cells predominated in both compartments, parenchyma and airways. This is in accordance with previous reports on extrapulmonary ALI and it differs from pulmonary ALI in which polymorphonuclear cells are the predominant cells infiltrating the lung [21], [22]. Although at the time point after sepsis (6 h) used in this study the lungs clearly presented inflammatory alterations, the lung function was not yet affected. We analysed the airways' responsiveness to methacholine by whole body plethysmography (Buxco) and found that the respiratory function was not significantly affected by CLP at this time in non-diabetic rats (data not shown). Thus, it seems that at this time point, the inflammatory process has been initiated at molecular and cellular level but it did not affect the respiratory function yet.

We found that although the diabetic animals with sepsis die earlier, they present milder lung inflammation than non-diabetics. This is in accordance with observations that during sepsis patients with diabetes are less likely to develop acute respiratory failure [12] and that septic-shock patients with a history of diabetes mellitus have a decreased risk of developing ALI or its more severe form ARDS (Acute Respiratory Distress Syndrome) compared with patients without diabetes [11]. Our results reported here shed light on the mechanisms involved in lung ‘protection’ in diabetics.

It has also been recently shown [4] that the inflammatory response is lower at the site of infection (peritoneal cavity) in diabetics. It was shown that rolling, adhesion, and migration of leukocytes were reduced in diabetic rats. The authors also show that the clearance of bacteria in the peritoneal cavity is impaired. In this situation it is easy to correlate the increased sepsis with the lower inflammation.

The reduced inflammatory lung inflammatory response in diabetic rats, seems to be restricted to the lung since diabetics present even increased renal dysfunction after sepsis, and there is no difference in cardiovascular, hepatic, haematological or metabolic dysfunction between diabetic and non-diabetic patients with sepsis [12].

Sepsis induced the expression of the lung-inducible prostaglandin syntase, COX2, in non-diabetic but not in diabetic rats. This suggested to us the possibility that diabetic rats have some problem in the signalling cascade that leads to gene expression. In fact, previous work by our group has shown that NFκB activation was impaired in diabetic lungs following LPS instillation [23]. In the present study we found that in AMs from diabetic rats with sepsis the NF-κB activation is impaired, as can be inferred from the reduced phosphorylation of the inhibitory protein IκB-α and the p65 subunit, which is responsible for gene transcription.

Signalling through TLRs requires the adaptor molecule MyD88 to be coupled to the receptor. The expression of MyD88 is inhibited by SOCS-1 [19] and we observed that, in our sepsis-induced ALI model, the alveolar macrophages from diabetic animals over-expressed SOCS-1 mRNA. At the same time, MyD88 mRNA was not detected in the diabetic rats. In addition, Serezani et al. [19] have shown that Gαi signalling-mediated cyclic AMP decrease inhibits SOCS-1 expression by enhancing SOCS-1 mRNA turnover.

The results presented suggest that during sepsis AMs from diabetic rats over-express SOCS-1 which inhibits the expression of MyD88, thus preventing TLR-mediated signal transduction, NFkB activation and therefore the transcription of inflammatory genes.

One limitation of this study is the fact that the lung inflammation was analysed at a single time point for reasons already explained and thus it is not possible to assume what would happen at later time points. Another limitation is the difficulty of extrapolating these findings to humans, as critically-ill patients are promptly treated with insulin to control the glycaemia.

The finding that reduced inflammation in diabetics with sepsis is related to impaired activation of NF-kB in alveolar macrophages unveils a novel mechanism that helps to explain the molecular basis for the lung ‘protection’ observed in diabetics with sepsis.
